# Assessment of positive selection across SARS-CoV-2 variants via maximum likelihood

**DOI:** 10.1371/journal.pone.0291271

**Published:** 2023-09-14

**Authors:** Carly Middleton, Laura Kubatko

**Affiliations:** 1 Department of Bioinformatics and Biostatistics, School of Public Health and Information Sciences, University of Louisville, Louisville, KY, United States of America; 2 Division of Biostatistics, College of Public Health, The Ohio State University, Columbus, OH, United States of America; 3 Department of Statistics, The Ohio State University, Columbus, OH, United States of America; 4 Department of Evolution, Ecology, and Organismal Biology, The Ohio State University, Columbus, OH, United States of America; Federal Medical Centre Abeokuta, NIGERIA

## Abstract

Study of the genome of the SARS-CoV-2 virus, particularly with regard to understanding evolution of the virus, is crucial for managing the COVID-19 pandemic. To this end, we sample viral genomes from the GISAID repository and use several of the maximum likelihood approaches implemented in PAML, a collection of open source programs for phylogenetic analyses of DNA and protein sequences, to assess evidence for positive selection in the protein-coding regions of the SARS-CoV-2 genome. Across all major variants identified by June 2021, we find limited evidence for positive selection. In particular, we identify positive selection in a small proportion of sites (5-15%) in the protein-coding region of the spike protein across variants. Most other variants did not show a strong signal for positive selection overall, though there were indications of positive selection in the Delta and Kappa variants for the nucleocapsid protein. We additionally use a forward selection procedure to fit a model that allows branch-specific estimates of selection along a phylogeny relating the variants, and find that there is variation in the selective pressure across variants for the spike protein. Our results highlight the utility of computational approaches for identifying genomic regions under selection.

## Introduction

COVID-19 was officially declared a global pandemic on March 11, 2020 [1]. By November 2021, the US death toll reached 753,000, with 46 million confirmed cases nationally [2]. The most transmissible SARS-CoV-2 variant at the time, Delta, was quickly replaced by the Omicron variant during the winter of 2021-2022 [3], including the BA.4 and BA.5 lineages which emerged in April of 2022 [4]. All variants remain a threat to public health today. With the recent rise in cases and deaths attributable to COVID-19, it is as important now as ever to be able to inform development of both legislation and policy for combating the pandemic.

In order to enable effective responses to this rapidly-evolving virus, understanding which regions of its genome are under positive selection is critical. Such knowledge can aid in providing a clearer picture of the evolution and mechanisms of the virus as well as assist in decision-making related to vaccine development. Though patterns of positive selection have previously been identified within the SARS-CoV-2 spike protein [5–7], the virus’s other structural proteins have yet to be widely tested, and a comparison of the extent to which positive selection is present across variants has, to our knowledge, not yet been made. The objective of this study is to test for the presence of positive selection within the spike, envelope, membrane, and nucleocapsid protein-coding regions of the SARS-CoV-2 genome and to compare the results across human and animal variants of the virus.

We tested for selection within the genomes of 11 SARS-CoV-2 human variants: Alpha, Beta, Delta, Gamma, Epsilon, Eta, Iota, Kappa, Lambda, Theta, and Zeta. We also tested the genomes of three SARS-CoV-2 variants which originated from animals: *Manis javanica, Rhinolophus sp*. and *Neovison vison*. Each of these animals has been either known or speculated to serve as a reservoir for SARS-CoV-2 and to have the potential of transferring the virus to and from humans [8, 9]. Furthermore, we tested for the presence of positive selection within regions of the SARS-CoV-2 genome that code for the spike protein, the envelope protein, the membrane protein, and the nucleocapsid protein because the structural proteins of SARS-CoV-2 play an integral role in the functionality and transmissibility of the virus. Though not examined here, we note that recent work has found that non-structural proteins may also be subject to selective pressure [6].

Our method of selection identification is a phylogenetic likelihood method. The method was implemented by applying PAML [10, 11], a collection of open source programs for phylogenetic analyses of DNA and protein sequences, to SARS-CoV-2 sequence data obtained from GISAID in 2021. Given genetic sequence data for which the phylogenetic relationships between sequences are known, the codeml program within PAML utilizes a standard codon substitution model to compute likelihoods associated with various values of *ω*, a selection parameter that indicates positive selection if greater than 1. We then compared values of *ω* across variants and loci. Results are presented from fitting several models executed by the codeml program within PAML and implications for SARS-CoV-2 evolution are discussed.

## Materials and methods

### Overview

The codon substitution model applied by PAML’s codeml program is the Goldman and Yang codon substitution model [12]. This model describes codon substitution as a Markov process parameterized by the nonsynonymous-to-synonymous mutation rate ratio, the transition-to-transversion rate ratio, and the steady-state frequencies of the codons within the genome. The nonsynonymous-to-synonymous mutation rate ratio, typically denoted as *ω*, is the model parameter of interest. It is estimated by codeml via a maximum likelihood approach.

Below we describe the acquisition process of genetic sequence data for this study as well as the method utilized to infer phylogenetic relationships between sequences. We then describe the process by which codeml was utilized to identify positive selection within and between SARS-CoV-2 variants.

### Data accession

1,238 SARS-CoV-2 sequences were obtained from the GISAID repository between the dates of June 6, 2021 and June 25, 2021. Sequences were collected from each of 11 human and four animal variants of the virus. For the *Rhinolophus sp*., *Manis javanica* and *Mus musculus* variants, all available sequences were downloaded. For all human variants and the *Neovison vison* variant, sequences were selected with the intention of promoting geographic diversity within the sample. To this end, sequences were sampled in rough proportion to the number available from each continent at the time of sampling. These proportions were only achievable for the Alpha, Beta and Delta datasets (Table 1). Within each continent, an effort was made to download sequences from a variety of countries as well as sequences that were submitted within 30 days of collection. Datasets were created by combining the downloaded sequences into a single file for each variant of the virus. Within each dataset, sequences were aligned using the multiple sequence alignment program MAFFT [13] with settings “–thread -1 –keeplength –add.”

**Table 1:** Acquired sequence data. Contents of each variant dataset. Official variant classifications (VOC, VUI, etc.) are accurate to the date of sequence download from GISAID.

Variant Name	Location First Discovered	Total Sequences	Sequence Count By Geographic Region					
			Europe	Asia	North America	Africa	South America	Oceania
VOC Alpha	United Kingdom	100	22	22	22	12	11	11
VOC Beta	South Africa	100	22	22	22	12	11	11
VOC Delta	India	100	22	22	22	12	11	11
VUI Epsilon	United States	100	0	1	98	0	1	0
VOC Eta	Nigeria	100	61	8	23	8	0	0
VOC Gamma	Brazil, Japan	100	14	0	57	0	29	0
VOI Iota	United States	100	25	25	25	2	16	7
VOI Kappa	India	100	30	56	10	0	0	4
VOI Lambda	Peru	100	19	0	36	0	45	0
VOI Theta	Philippines	100	18	71	7	0	0	4
VOI Zeta	Brazil	100	12	2	57	0	28	1
*Neovison vison*		102	64	38	0	0	0	0
*Rhinolophus sp*.		13	0	13	0	0	0	0
*Manis javanica*		19	0	19	0	0	0	0
*Mus musculus*		4	0	3	1	0	0	0

The data collection process resulted in 15 variant datasets, most of which consisted of 100 SARS-CoV-2 sequences. For each dataset, the variant name, number of sequences, geographic distribution of sequences, and location at which the variant was first discovered are listed in Table 1. We note that our sampling strategy was not designed to account for founder effects or sample acquisition bias, but rather to obtain samples in the most geographically comprehensive manner possible given the sequences available in GSAID at the time of data collection.

### Statistical methodology

#### Inference of phylogenetic relationships between sequences

Given a set of sequences, the open source program IQ-TREE 2 [14] uses maximum likelihood estimation to produce likelihoods associated with various candidate phylogenetic trees relating the sequences. The program then chooses a maximum likelihood estimate of the dataset’s true phylogenetic tree. IQ-TREE 2’s ModelFinder Plus utility iterates through up to 286 possible DNA substitution models and then chooses the best fit model according to the associated values of AIC (Akaike information criterion), BIC (Bayesian information criterion), or AICc (second-order Akaike information criterion). In this study, IQ-TREE 2’s ModelFinder Plus utility with the AIC option was utilized. The result was one maximum likelihood estimate of the evolutionary relationships between sequences for each variant dataset, each in the form of a phylogenetic tree. These estimates were then specified in codeml for the remainder of the analysis.

#### Selection identification within variants

The primary task of this study is to test for positive selection within SARS-CoV-2 variant genomes. This task was completed by applying codeml’s M0, M3, and M8 models to each dataset; each of these models estimate *ω* but operate under different assumptions. The M0 model assumes that the entire protein-coding region of interest can be described by a single value of *ω*. The greatest utility of the M0 model is that it provides a global “snapshot” of whether or not selection is present overall in a protein-coding region.

The M3 model assumes that each codon along the protein-coding region falls into one of K site classes for a user-specified K, each which can be described by a value of *ω*. A likelihood function is maximized in order to obtain the K estimates of *ω* for each site class as well as their associated probabilities. For each value of *ω*, the associated probability estimate is the proportion of codon sites within the protein-coding region that have the particular value of *ω*. The M3 model indicates the proportion of the protein-coding region that is under strong selection versus weak selection. The model can also identify whether or not subsets of the region are under selection, even in cases when selection cannot be identified within the region as a whole.

The M8 model assumes that there is only one value of *ω* within the protein coding region which is indicative of positive selection, but various values of *ω* which do not indicate positive selection. The model calculates the probability that *ω* > 1 for each site, as well as the most likely value of *ω* for sites in this category. The *ω* values of the remaining sites are assumed to follow a Beta distribution, and the program estimates the shape parameters *α* and *β* of this Beta distribution. M8 can identify the proportion of sites that are under positive selection versus negative selection within a region.

codeml’s M0, M3, and M8 models were fit for the spike, envelope, membrane, and nucleocapsid protein-coding regions within each SARS-CoV-2 variant dataset; model results for *Mus musculus* are not discussed due to this dataset’s small sample size. For all three models, the seqtype = 1, CodonFreq = 2, and model = 0 options within codeml were specified in order to indicate a codon model with equal codon frequencies and one *ω* ratio for all branches. Initial values of *κ* = 2 and *ω* = 1 were specified for maximum likelihood estimation. The NSsites option was set to 0, 3, and 8 for the M0, M3, and M8 models, respectively. For the M3 model, the ncatG option was used in order to specify K. M3 was fit for both *K* = 3 and *K* = 4 for all datasets. In some cases, PAML’s reported parameter estimates were on the boundary of the parameter space, indicating difficulty in convergence for the algorithm, possibly due to flatness of the likelihood surface. We note that it is also possible that the likelihood surface is relatively flat even when a valid estimate of *ω* is obtained, and this is difficult to assess without an estimate of error in the estimate.

#### Selection identification between variants

**Fig 1:**
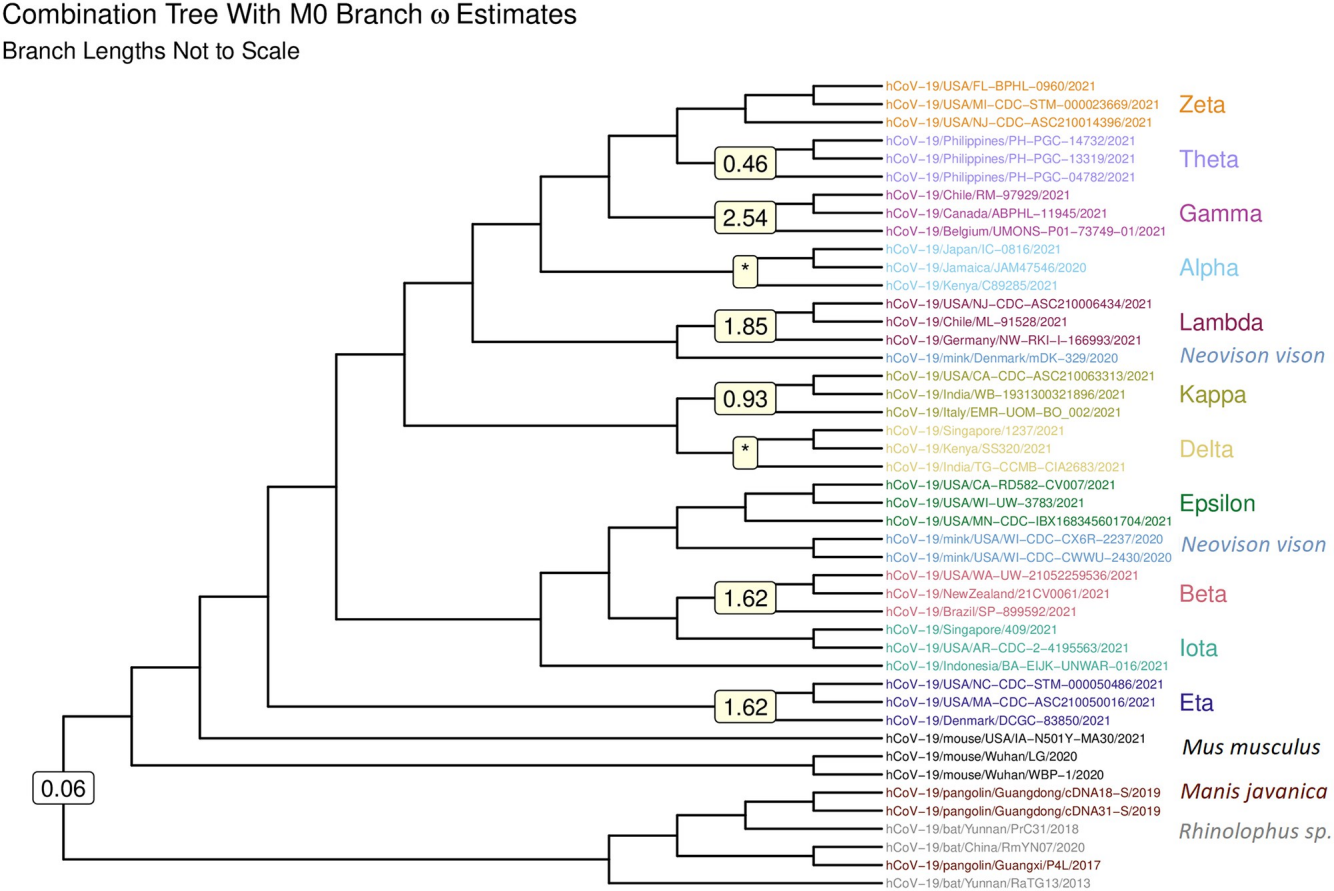
Phylogeny for the combination dataset. Phylogenetic tree estimated by maximum likelihood via IQ-TREE 2 [14]. Branch lengths are not drawn to scale. Values on the branches are the branch-specific *ω* estimates resulting from the forward selection. Branch-specific estimates for the Alpha and Delta clades, denoted with asterisks, were not obtained due to failure of the M0 algorithm to reach convergence. This visualization was created with the R packages ggtree [16] and treeio [17].

A combination dataset was created by merging three randomly selected spike protein sequences from each dataset into a single sequence file. The phylogenetic tree for this dataset was then estimated using IQ-TREE 2 (Fig 1). The tree was rooted using outgroup rooting with the software FigTree [15] in order to classify the ancient bat and pangolin sequences as outgroups in comparison to the more diverged sequences.

We wish to determine whether or not the selection parameter *ω* differs significantly between monophyletic clades of the combination dataset. This determination can be made with M0, which allows the user to generate separate estimates of *ω* for each branch along a phylogenetic tree by specifying model = 2. We employed a forward selection method for obtaining a combination tree with one *ω* value for each monophyletic clade. Likelihood ratio tests were used in order to justify decisions to retain or remove predicted *ω* values at each step of the forward selection.

The results of step 1 of the forward selection are shown in Table 2. The null hypothesis for step 1 was that *ω* does not differ between the clade of interest and the rest of the tree, and the alternative hypothesis was that *ω* differs between the clade of interest and the rest of the tree. The Gamma clade showed the strongest evidence of a differing *ω* (*p* = 8.11 x 10^−7^). The conclusion from step 1 was that the tree can be described by at least two distinct branch *ω* values: one for the Gamma clade and one for the rest of the tree. The latter statement became the null hypothesis of step 2. The forward selection was repeated until no null hypotheses were rejected at a 5% significance level.

**Table 2:** **(a)** p-values from the step 1 likelihood ratio tests of the forward selection, and **(b)** summary of steps in the forward selection procedure.

(**a**)		
*H*_*a*_		p-value
Alpha’s clade different		9.64 x 10^−5^
Beta’s clade different		1.13 x 10^−2^
Delta’s clade different		2.59 x 10^−3^
**Gamma’s clade different**		**8.11 x 10** ^**−7**^
Eta’s clade different		7.05 x 10^−3^
Epsilon’s clade different		6.82 x 10^−2^
Kappa’s clade different		9.80 x 10^−2^
Lambda’s clade different		3.18 x 10^−4^
Theta’s clade different		6.66 x 10^−2^
Zeta’s clade different		8.26 x 10^−2^
(**b**)		
Step	Clade Added	p-value
1	Gamma	8.11 x 10^−7^
2	Alpha	1.46 x 10^−7^
3	Lambda	1.42 x 10^−5^
4	Delta	1.35 x 10^−5^
5	Beta	4.85 x 10^−5^
6	Eta	4.92 x 10^−5^
7	Kappa	4.31 x 10^−5^
8	Theta	2.48 x 10^−3^

## Results

### Selection identification within variants

**Fig 2:**
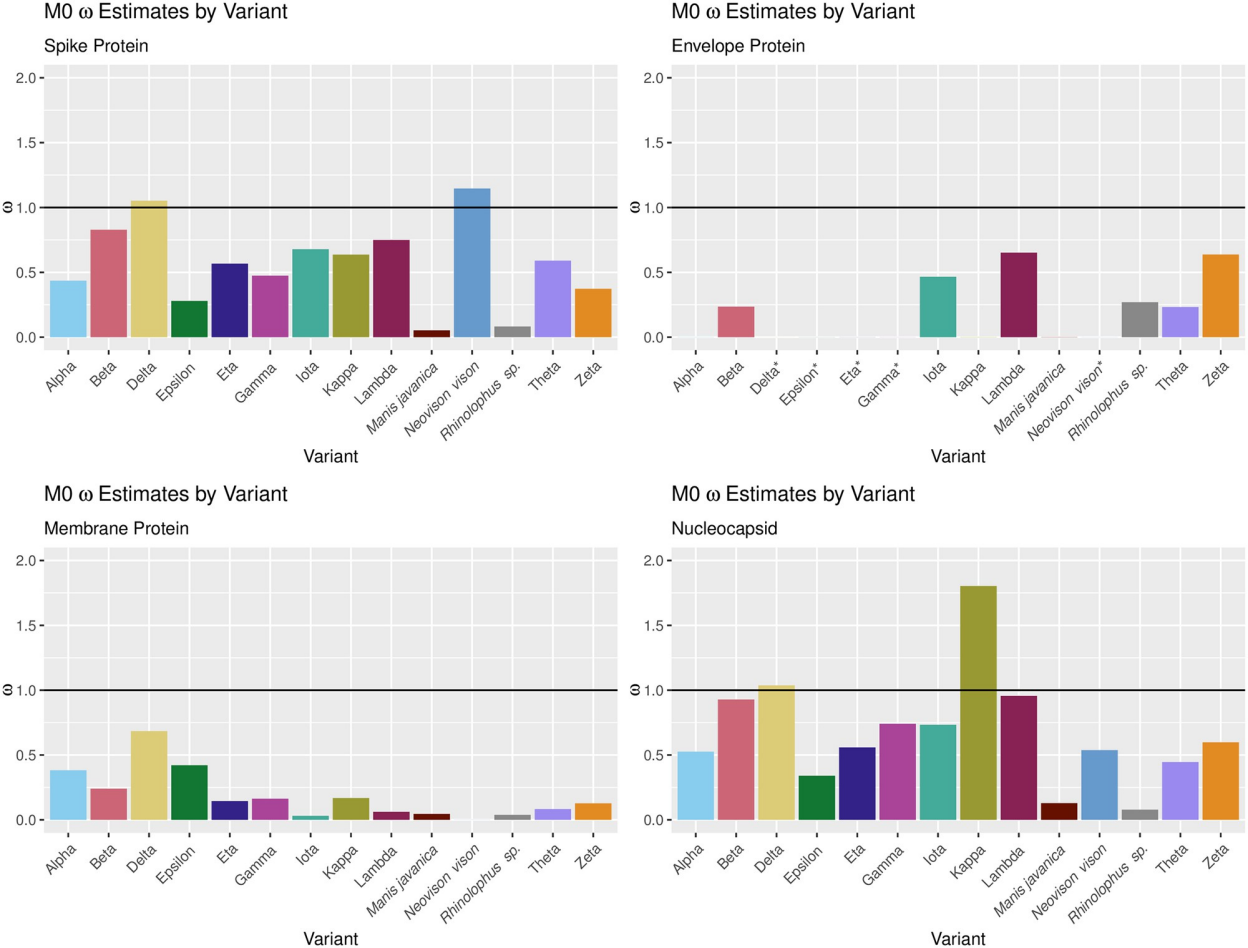
Within-variant *ω* estimates: M0 model. *ω* estimates for each variant yielded by PAML’s M0 model at the spike, envelope, membrane, and nucleocapsid protein-coding regions. For the Delta, Epsilon, Eta, Gamma, and *Neovison vison* variants within the envelope protein-coding region, the algorithm for estimating *ω* failed to converge (denoted by an asterisk in the figure).

M0 estimates of *ω* for each variant within the spike, envelope, membrane, and nucleocapsid protein-coding regions are shown in Fig 2. Within the spike protein-coding region, the Delta and *Neovison vison* variants are associated with an *ω* value greater than 1. This suggests that positive selection was present within these two variants at the locus that codes for the spike protein. Within the nucleocapsid protein-coding region, the Delta and Kappa variants are associated with *ω* values greater than 1; the implication is the same. Within the membrane protein-coding region, positive selection is not identified for any of the variants. Within the envelope protein-coding region, the algorithm for estimating *ω* did not converge for the Delta, Epsilon, Eta, Gamma, and *Neovison vison* variants, and none of the other variants led to an estimated *ω* greater than 1.

**Fig 3:**
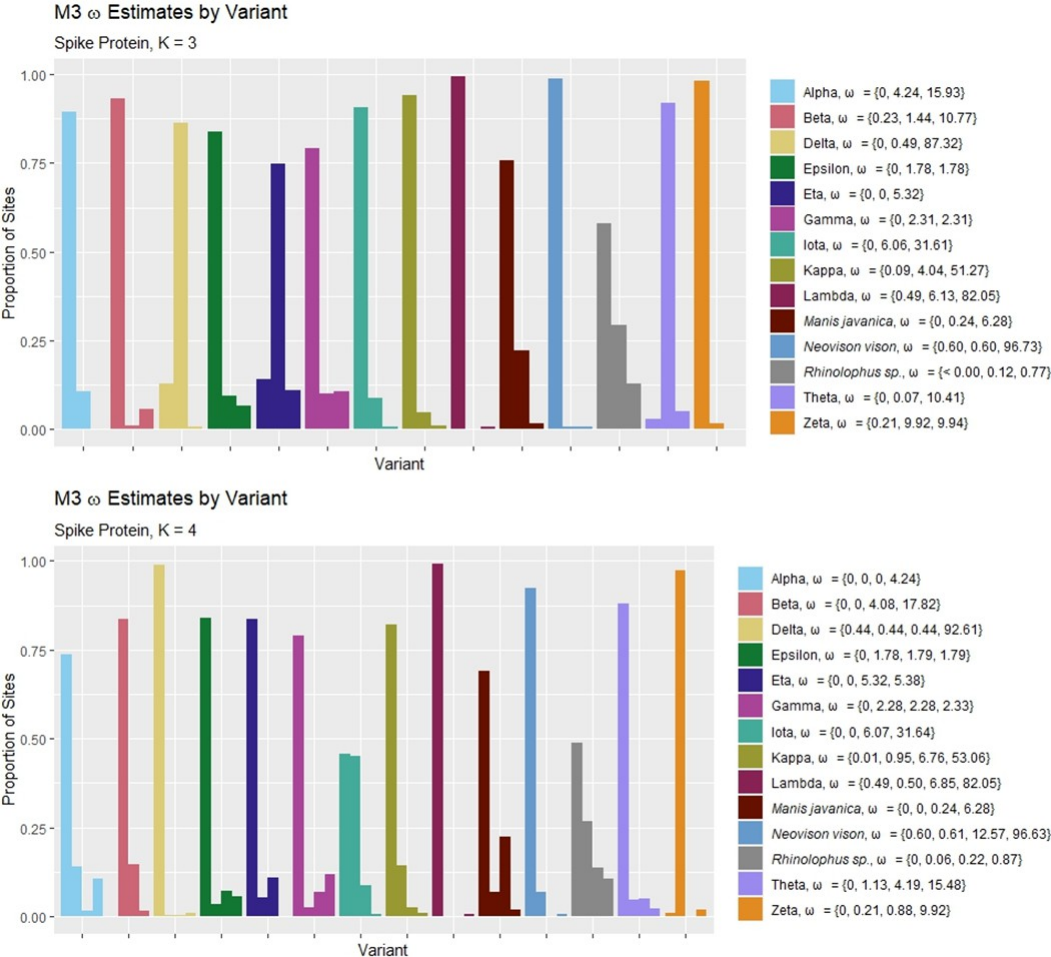
Within-variant *ω* estimates: M3 model. Parameter estimates and associated probabilities yielded by PAML’s M3 model for the spike protein-coding region with both *K* = 3 and *K* = 4 site categories. For each variant, the legend lists, in order, the *K* *ω* estimates associated with each of the *K* probability estimates shown from left to right on the graph. For example, for the Alpha variant when *K* = 3, M3 estimates that 89.30% of sites evolved with *ω* = 0, 10.71% of sites evolved with *ω* = 4.24, and no sites evolved with the largest *ω* value estimated (15.93).

Fig 3 displays the results of applying PAML’s M3 model to the spike protein-coding region. For both *K* = 3 and *K* = 4, it can be seen that within variants, one estimated value of *ω* typically has an associated probability that is much greater than the others. Judging from the estimates of *ω* in the category that has the greatest estimated proportion of sites, both the *K* = 3 and *K* = 4 results suggest that most of the spike protein was not under positive selection before the time of data collection. However, there is evidence that for some of the variants, some sites in the alignment may have been under positive selection. For example, for the Alpha variant and for *K* = 3, it is estimated that approximately 10% of the sites evolved with an *ω* value of 4.24. Many other variants show indications of positive selection for 5%–15% of the sites in the protein coding region.

**Fig 4:**
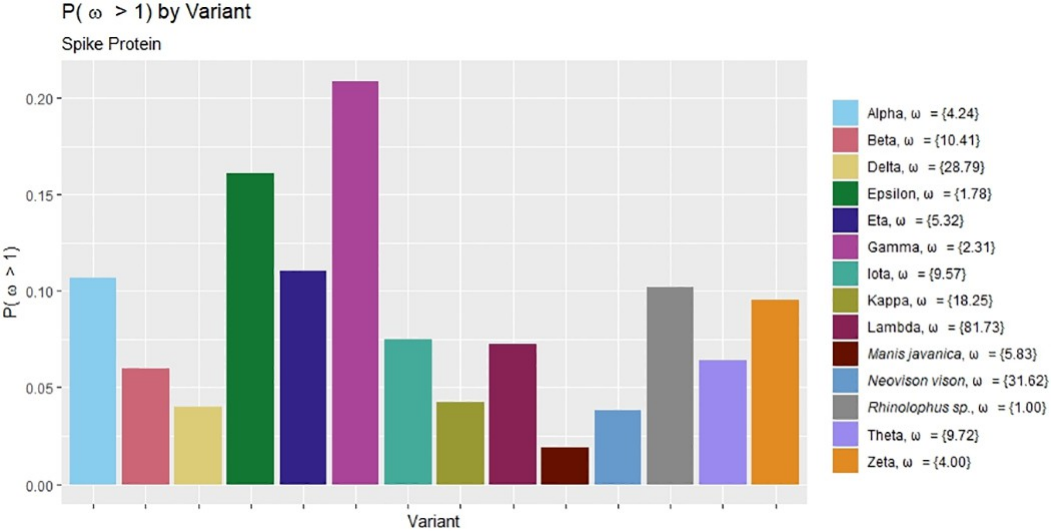
Within-variant *ω* estimates: M8 model. Conditional probabilities of parameter estimates yielded by PAML’s M8 model for the spike protein-coding region under the *ω* > 1 assumption. The legend lists the *ω* estimate associated with each conditional probability. For example, for the Alpha variant, M8 predicts that *P*(*ω* > 1) = 0.11, and that if this is the case, *ω* = 4.24.

PAML’s M8 model estimates a single value of *ω* for each protein-coding region of interest under the assumption that *ω* > 1, and a Beta-distributed range of probabilities for *ω* values under the assumption that *ω* < 1. Results for the *ω* > 1 assumption are shown in Fig 4. The estimated proportion of sites that evolved under positive selection is less than 30% for all variants, suggesting that selection has occurred for only a limited region of the spike protein.

### Selection identification between variants

The results of our forward selection procedure for determining branch-specific *ω* values for the phylogeny estimated from the combination dataset are shown in Fig 1. With the exception of the Kappa and Theta variants, all of the branch-specific estimates of *ω* are greater than 1, suggesting positive selection has acted on the variants for which distinct values were selected in the forward selection procedure (Gamma, Lambda, Beta, and Eta). As in the M0 model, the estimates for the Alpha and Delta variants reached the upper limit set by the algorihtm, suggesting problems with convergence of the algorithm. Interestingly, the Beta and Eta variants are associated with similar *ω* values, suggesting that they may have evolved under similar constraints.

## Discussion

The major finding from this study is that PAML’s codon substitution model produced *ω* estimates suggestive of positive selection for several variants within several protein-coding regions: Delta within the spike protein and nucleocapsid, *Neovison vison* within the spike protein, and Kappa within the nucleocapsid. It is not surprising that Delta has been under selective pressure during the second year of the pandemic, given the variant’s high transmissibility and virulence. Though the Kappa variant is not currently of major concern due to its relatively low transmissibility and lack of virulence [18], the suggestion that the American mink variant was under selective pressure could be concerning because SARS-CoV-2 has been proven to transfer frequently between humans and minks [8, 19]. Future research may be necessary in order to address the question of how to protect humans from this variant. In addition, we have focused here on detecting selection within regions of the virus that code for structural proteins. Recent work [6] suggests that regions coding for non-structural proteins may also be subject to selective pressure.

One possible driver of our results is the sampling scheme utilized. In order to obtain geographic representation within the sample, for each variant we attempted to download 22 sequences from each of Europe, Asia and North America, 12 sequences from Africa, and 11 sequences each from South America and Oceania; however, this ratio was not achieved for most variants due to limited sequence availability at the time of sampling. The task of deciding on an optimal sampling scheme for this study was not an easy one; though many different approaches were possible including use of a simple random sampling scheme, an alteration of the 22:12:11 ratio, or even to perform separate analyses by continent, our strategy was to focus on geographic representation. One future direction for this study could be to repeat the analysis on different datasets obtained from one of the above sampling schemes and then compare the results to our results yielded by the original datasets.

A small portion of missing base pairs was present in many of the sequences. In genetic sequence data, missing data can occur for several reasons, which may include issues in sample preparation or genotyping. For data obtained by next-generation sequencing (NGS), it is common for portions of a sequence to be either not sequenced or sequenced at very low read depth [20], which can result in missing data. In the sequences downloaded, some variants had more missing base pairs than others (for example, the Theta dataset had a much larger proportion of missing data than any other variant). One possible solution could be to impute missing base pairs before performing the analysis. Though methodology has previously been proposed for imputation of sequence data from the human genome [21, 22], alteration of such methods may be necessary for application to viral sequences.

While we have focused on the use of the PAML software to assess selection, other methods are possible. For example, the ADAPTSITE software [23] uses a parsimony-based approach to identification of sites under selection. Whether such an approach is appropriate for viruses that are known to have a relatively high evolutionary rate (and to therefore be subject to multiple hits) is debatable. Another option is use of the MrBayes software [24] to obtain estimates of *ω* as well as the proportion of sites under selection. While MrBayes has the advantage of providing information about the entire posterior distribution of *ω* (and thus an assessment of the estimation error, which PAML does not provide), it also requires specification of prior distributions, monitoring of convergence (which might be time-intensive for the dataset sizes considered here), and assessment of prior sensitivity.

This study demonstrates how the maximum likelihood method implemented by PAML can be used to detect regions of the SARS-CoV-2 genome that are undergoing positive selection. One practical extension of this study would thus be to use this method to carry out real-time surveillance in an attempt to rapidly detect regions of the genome that are evolving subject to selective pressure. For example, datasets that include newly sequenced samples could be obtained daily from GISAID, and *ω* values could be estimated from these datasets. Changes in the values of *ω* could indicate novel evolutionary pressures, perhaps identifying locations in the genome that might become functionally important. While the analyses used by PAML are computationally intensive, and thus limited to smaller samples (the sample sizes used in this study are near the limit for efficient analysis), development of an automated pipeline and the analysis of differing samples with similar compositions could be used to efficiently gain broader insights.

## Conclusion

We used PAML to understand the selective pressures underlying evolution of the SARS-CoV-2 virus. Overall, there is evidence for positive selection in a small proportion (5-15%) of sites in the protein-coding region of the spike protein for several of the variants. The other variants do not show a strong signal for positive selection overall, though there is some indication of positive selection in the Delta and Kappa variants for the nucleocapsid protein. Our forward selection procedure for fitting branch-specific estimates of *ω* to a phylogeny consisting of all variants considered shows significant variation in the values of *ω* among the variants. Collectively, these results highlight the potential of computational tools for fitting evolutionary models to provide important insights in the evolutionary processes involved in SARS-CoV-2.

A strength of using PAML for such analyses is the range of models it employs. For example, the M0 model allows for a “snapshot” of the selective pressure acting on a protein-coding region as a whole, while models like M3 and M8 allow for estimation of selective pressure at subsets of sites in the genetic sequence. In addition, PAML’s ability to estimate branch-specific values of *ω* allows an examination of how selection might vary across the sequences included in a larger phylogeny. The range of approaches together provide a useful set of tools to understand the evolutionary processes underlying the evolution of the SARS-CoV-2 virus.
